# Gall Bladder Tumour, Choledochal Cyst and an Anomalous Pancreatico-Biliary Junction

**DOI:** 10.1155/1995/12618

**Published:** 1995

**Authors:** T. F. Toufeeq Khan, Zaheer A. Sherazi, Yan Yew Tan

**Affiliations:** 1 Department of Surgery School of Medical Sciences Universiti Sains Malaysia Malaysia; 2 Department of Radiology School of Medical Sciences Universiti Sains Malaysia Malaysia; 3 Department of Surgery Hospital Universiti Sains Malaysia School of Medical Sciences Universiti Sains Malaysia Malaysia; 4 Department of Surgery Hospital Universiti Sains Malaysia 16150 Kubang Kerian Kelantan Malaysia

## Abstract

This report highlights the association of a tumour in an acalculous gall bladder with an anomalous pancreatico-biliary junction (PBJ) and a type IVa choledochal cyst. Cholecystectomy and Rouxen- Y hepatico-jejunostomy (RHJ) was performed after division of the common bile duct (CBD) and excision of the dilated segment. The details of the case are presented and the role of an abnormal PBJ in gall bladder carcinogenesis is discussed.


*HPB Surgery*, 1995, Vol. 8, pp. 185–186
Reprints available directly from the publisher
Photocopying permitted by license only

© 1995 Harwood Academic Publishers GmbH
Printed in Malaysia

### Gall Bladder Tumour, Choledochal Cyst and an Anomalous Pancreatico-Biliary Junction

T. F. TOUFEEQ KHAN[1], ZAHEER A. SHERAZI[2] and YAN YEW TAN[3]

[1] Department of Surgery, [2] Department of Radiology, [3] Department of Surgery Hospital Universiti Sains Malaysia, School of Medical Sciences, Universiti Sains Malaysia

This report highlights the association of a tumour in an acalculous gall bladder with an anomalous pancreatico-biliary junction (PBJ) and a type IVa choledochal cyst. Cholecystectomy and Roux-en-Y hepatico-jejunostomy (RHJ) was performed after division of the common bile duct (CBD) and excision of the dilated segment. The details of the case are presented and the role of an abnormal PBJ in gall bladder carcinogenesis is discussed.

KEY WORDS: Anomalous pancreatico-biliary junction gall bladder tumour choledochal cyst.

### INTRODUCTION

An abnormal PBJ occurs when the pancreatic duct unites with the distal CBD proximal to the duodenal sphincter. This results in the continous reflux of pancreatic enzymes into the biliary tree. The repeated inflammation eventually leads to mucosal changes in the biliary tract. Recently, the abnormal PBJ has been shown to be associated with malignant changes in the gall bladder[1]. In patients with an abnormal PBJ, the aim of surgery is to prevent pancreatic enzyme reflux by disconnecting this junction from the biliary tree and to perform a bilio- enteric anastomosis. We report a case of a tumour within an acalculous gall bladder with an abnormal PBJ. The aetiological correlation in the light of present concepts is discussed.

### CASE REPORT

A 49 year old housewife was admitted to Hospital Universiti Sains Malaysia with acute right upper abdominal pain and vomiting of 3 days. She was admitted with similar complaints and jaundice 2 years ago when ultrasound revealed a soft tissue mass in the gall bladder. She was offered surgery but defaulted.

On examination, mild tenderness was elicited on palpation in the upper abdomen. She was non-icteric with normal liver function tests. An ultrasound scan revealed a hyperechoic soft tissue mass without acoustic shadowing filling the gall bladder (Fig. 1 a & b). Endoscopic retrograde cholangio-pancreatography (ERCP) showed a grossly dilated cystic duct and a gall bladder with multiple filling defects. The CBD appeared dilated and was overlapped by the large calibre cystic duct. The proximal right hepatic duct was also dilated and an abnormal PBJ was evident (Fig. 2). A diagnosis of carcinoma of gall bladder associated with an abnormal PBJ and type IVa choledochal cyst was made. Laparotomy confirmed a soft tissue tumour filling the gall bladder. The cystic duct measuring 1.2 cm was entering the anterior wall of the dilated CBD.

Per-operative cholangiography revealed essentially the same findings as ERCP. A cholecystectomy and an excision of the supraduodenal CBD was performed. The lower end was closed behind the duodenum and a 60 cm Roux loop was used to fashion a hepatico-jejunostomy. She made an uneventful recovery and was discharged ten days after surgery. Histology revealed a villous adenoma with areas of metaplasia and some atypical cells without stromal involvement. She remains symptom free at 14 months after surgery.

### DISCUSSION

The abnormal PBJ is the junction of the terminal bile duct and pancreatic duct proximal to the duodenal sphincter and enters the duodenum as a single duct or a long

*Address for correspondence:* Department of Surgery, Hospital Universiti Sains Malaysia, 16150 Kubang Kerian, Kelantan, Malaysia

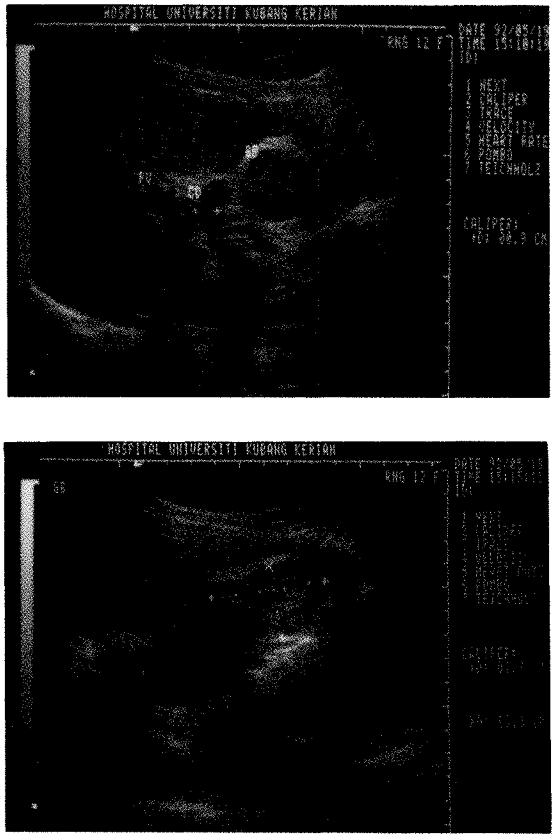

**Figure 1 a, b** Ultrasonogram of the gall bladder, sagittal (a) and axial scans (b) showing a hyperechoic soft tissue mass without acoustic shadowing filling the lumen.

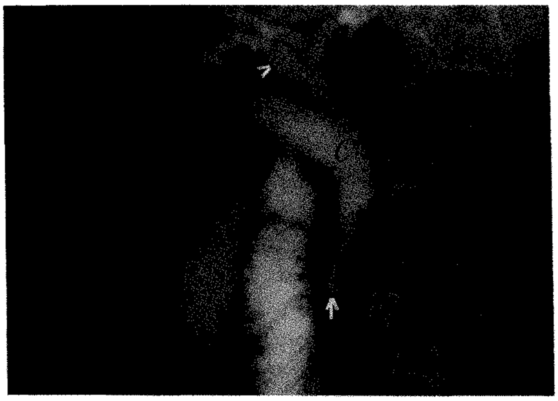

**Figure 2** ERCP demonstrating the abnormal pancreaticobiliary junction (arrow) and the dilated cystic duct. Cystic dilation is seen in the area of the common bile duct (C) and the right hepatic duct (arrow head).

common channel. Being void of sphincteric control, pancreatic enzymes can reflux into the biliary tree and gall bladder. It has been proposed that the abnormal PBJ may be responsible for the development of gall bladder cancer[1-5]. The aetiological role of the abnormal PBJ in gall bladder tumour seems likely in our patient especially in the absence of gall stones and considering her younger age group. Twenty-five per cent of gall bladder cancers arise in acalculous gall bladders[6]. The level of pancreatic enzymes in the gall bladder is higher than in the bile ducts in these patients because of its concentrating ability. The resulting long standing inflammation induces mucosal changes within the gall bladder and the biliary tract. The association of metaplasia with dysplasia and carcinoma is strengthened by the fact that these latter changes are only seen in areas of metaplasia[7]. The association of metaplasia with gall bladder cancer has also been suggested by others[8,9]. There has been increasing evidence of coexisting pancreatico- biliary anomalies in patients with choledochal cysts[10]. This infers a collection of developmental defects in the pancreatico-biliary ducts rather than a single discrete lesion as demonstrated in our patient.

In gall bladder tumours without gall stones an abnormal PBJ must be vigorously sought for by ERCP or per-operative cholangiography. If discovered, in addition to cholecystectomy, we advocate separation of the biliary and pancreatic systems by division of the bile duct, to prevent further long term complications.

### REFERENCES

1. Kimura, K., Ohto, M., Saisho. H., *et al.* (1985), Association of gall bladder carcinoma and anomalous pancreatico-biliary ductal union. *Gastroenterology* **85,** 1258–65.
2. Kozuka, S., Kurashina, M., Tsubone, M. *et al.* (1984), Significance of intestinal metaplasia for the evolution of cancer in the biliary tract. *Cancer* **54,** 2277–85.
3. Sameshima, Y., Uchimura, M., Muto, Y. *et al.* (1978), A case of anomalous arrangement of the pancreatico-biliary duct with papillary adenoma of the gall bladder. *Jpn, J., Gastroenterol* **15,** 909–15. (In Japanese)
4. Sameshima, Y., Uchimura, M., Muto, Y. *et al.* (1987), Co-existent carcinoma in congenital dilation of the bile duct and anomalous arrangement of the pancreatico-bile duct. *Cancer* **60,** 1883–90.
5. Miyazaki, K., Date, K., Imamura, S. *et al.* (1989), Familial occurrence of anomalous pancreatico-biliary duct union associated with gall bladder neoplasms. *Am. J. Gastroenterol* **84,** 176–82.
6. Jones, R. S. (1990), Carcinoma, of the Gall Bladder. *Surg. Clin. North Am.* **70,** 1419–28.
7. Laito, M. (1983), Histogenesis of epithelial neoplasms of human gall bladder I and II. *Pathol Res. Pract.* **178,** 51–66.
8. Jarbi, O., Lauren, P. (1967) Intestinal metaplasia in the mucosa of the gall bladder and common bile duct. *Ann. Med. Exp. Fenn.* **45,** 213–23.
9. Matsumine, T., Kubota, Y., Yamaoka, I. *et al.* (1978), A histopathological study of the gall bladder carcinoma: With reference to intestinal metaplasia (duodenization) of the cholecystic mucosa. *J. Jpn. Soc. Clin. Surg.* **39,** 927–34. (In Japanese)
10. Nagorney, D. M., McIlrath, D.C., Adson, M.A. (1984), Choledochal cysts in adults: Clinical management. *Surgery* **96,** 656–63.

